# *Vibrio cholerae* Proteome-Wide Screen for Immunostimulatory Proteins Identifies Phosphatidylserine Decarboxylase as a Novel Toll-Like Receptor 4 Agonist

**DOI:** 10.1371/journal.ppat.1000556

**Published:** 2009-08-21

**Authors:** Ann Thanawastien, Wagner R. Montor, Joshua LaBaer, John J. Mekalanos, Sang Sun Yoon

**Affiliations:** 1 Department of Microbiology and Molecular Genetics, Harvard Medical School, Boston, Massachusetts, United States of America; 2 Harvard Institute of Proteomics, Harvard Medical School, Cambridge, Massachusetts, United States of America; 3 Department of Microbiology and Immunology, University of Otago, Dunedin, New Zealand; 4 Department of Microbiology, College of Medicine, Yonsei University, Seodaemun-gu, Seoul, Korea; University of Pennsylvania, United States of America

## Abstract

Recognition of conserved bacterial components provides immediate and efficient immune responses and plays a critical role in triggering antigen-specific adaptive immunity. To date, most microbial components that are detected by host innate immune system are non-proteinaceous structural components. In order to identify novel bacterial immunostimulatory proteins, we developed a new high-throughput approach called “EPSIA”, ***E***xpressed ***P***rotein ***S***creen for ***I***mmune ***A***ctivators. Out of 3,882 *Vibrio cholerae* proteins, we identified phosphatidylserine decarboxylase (PSD) as a conserved bacterial protein capable of activating host innate immunity. PSD in concentrations as low as 100 ng/ml stimulated RAW264.7 murine macrophage cells and primary peritoneal macrophage cells to secrete TNFα and IL-6, respectively. PSD-induced proinflammatory response was dependent on the presence of MyD88, a known adaptor molecule for innate immune response. An enzymatically inactive PSD mutant and heat-inactivated PSD induced ∼40% and ∼15% of IL-6 production compared to that by native PSD, respectively. This suggests that PSD induces the production of IL-6, in part, via its enzymatic activity. Subsequent receptor screening determined TLR4 as a receptor mediating the PSD-induced proinflammatory response. Moreover, no detectable IL-6 was produced in TLR4-deficient mouse macrophages by PSD. PSD also exhibited a strong adjuvant activity against a co-administered antigen, BSA. Anti-BSA response was decreased in TLR4-deficient mice immunized with BSA in combination with PSD, further proving the role of TLR4 in PSD signaling *in vivo*. Taken together, these results provide evidence for the identification of *V. cholerae* PSD as a novel TLR4 agonist and further demonstrate the potential application of PSD as a vaccine adjuvant.

## Author Summary

Innate immune responses are the first line of defense and involve the early recognition of pathogenic microorganisms. Furthermore, these early innate responses can help shape and influence the development of more specific adaptive immune responses. One way that innate immunity is triggered is by activation of TLRs, or Toll-like Receptors. TLRs recognize a wide spectrum of microbes by binding to pathogen-associated molecular patterns (PAMPs), which are conserved microbial products. Here, we have used a high-throughput method to understand more about how a pathogen can trigger early innate immune responses and also how these early responses to infection can influence the adaptive, more specific, immune response. This technique can also be utilized for adjuvant discovery which is important in vaccine development since different adjuvants can induce or enhance different kinds of immune responses to a particular antigen. Using this method, we identified a novel bacterial protein that activates a TLR and further characterized its role as an adjuvant. Identifying the TLRs, their ligands, and the signal transduction events that they initiate has provided insight into our understanding of how the immune response to infection begins, and how these factors also collectively influence the adaptive immune response.

## Introduction

Innate and adaptive immunity are two arms of the immune system that help defend against invading microbes [1],[2]. Innate immunity provides immediate defense against infection in a non-specific manner and also influences the antigen-specific adaptive immune response [3],[4]. The innate response to microbes involves the recognition of conserved microbial products, collectively termed pathogen-associated molecular patterns (PAMPs) by specific host receptors [2]. Germ-line encoded Toll-Like Receptors (TLRs) are the best characterized of these receptors [2],[5]. To date, 13 TLRs have been identified in mice, and a few of their cognate ligands include lipopolysaccharide (LPS) [6], peptidoglycan [7], diacyl- or triacyl-lipopeptide [8], dsRNA [9], unmethylated CpG DNA motifs [10], and flagellin, a subunit of bacterial flagella [11],[12]. When TLRs that are either surface-exposed or located in the endosomal membrane bind PAMPs, signal transduction events are activated leading to proinflammatory cytokine production [1]. The proinflammatory response induced by TLR activation can lead to active clearance of pathogens and an enhancement of the adaptive immune response.

Genome-wide approaches [13] have defined bacterial factors that cause, for example, cytotoxicity after expression in yeast [14], or the host factors that modulate bacterial intracellular replication [15],[16]. However, a comprehensive screen of the predicted proteome of a bacterial organism for immune activating proteins has not been previously reported [13]. Comprehensive genome-wide screening for bacterial proteins that elicit innate immune responses has been limited by two factors. Firstly, bacterial genomes encode for thousands of proteins and this necessitated the development of a resource that allows high-throughput purification or expression of individual proteins before screening can take place. Secondly, because evolutionary pressure can readily select for changes in amino acid sequence, proteins have been generally thought to have lost conserved motifs that might be recognized by the innate immune system. To date, only flagellin and membrane-anchored lipoproteins were reported to contain conserved motifs that are recognized by host TLRs [11],[17],[18],[19],[20]. Interestingly, bacterial flagellins were also determined to have robust adjuvant activity enhancing specific T-cell responses against co-administered antigens *in vivo* [21],[22]. Therefore, identification of new bacterial proteins that induce the host innate immunity will broaden our understanding of host responses to bacterial infection and provide an opportunity to develop new vaccine adjuvants.

Recently, we reported the production of a complete full-length open reading frame (ORF) expression library for the pathogenic bacterium *V. cholerae* [23]. In the same work, we showed that flagellins (FlaD and FlaC) synthesized from each of their ORF expression clones by *in vitro* transcription/translation are capable of eliciting NF-κB activation in A549 human lung epithelial cells [23]. This suggests that proteins produced by this system can be used for high-throughput screening for other protein activators of host innate immunity. In this study, we developed EPSIA technology as a high-throughput proteome-wide screen for stimulators of host innate immune system. Using *V. cholerae* as a test organism, we identified numerous new proinflammatory proteins and characterized the proinflammatory signaling mechanism induced by one of these protein hits, phosphatidylserine decarboxylase (PSD) that stimulated murine macrophage cells with the greatest potency. PSD activated mouse macrophages to secrete proinflammatory cytokines in a TLR4-dependent, MyD88-dependent manner, and full induction required a processed, enzymatically active PSD. Moreover, PSD was shown to act as a robust adjuvant when co-administered with an inert protein antigen, bovine serum albumin (BSA). Our results reveal the versatility of the EPSIA approach for uncovering new microbial activators of host innate immune responses.

## Results

### *V. cholerae* phosphatidylserine decarboxylase (PSD) is identified as an immunostimulatory protein from a high-throughput proteome-wide screening

**Figure 1:**
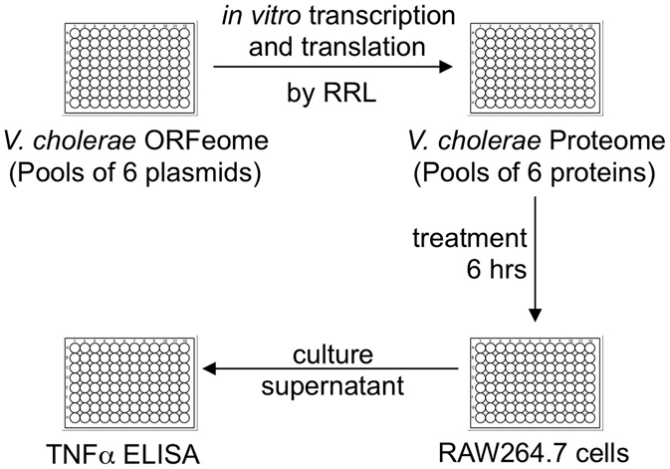
Screening of *V. cholerae* proteome library for immunostimulatory proteins. Proteome library was prepared from the ORF expression plasmid library by *in vitro* protein synthesis. Six plasmids were placed in each well. After being diluted 4-fold in PBS, rabbit reticulocyte lysate (RRL) mixtures containing proteins were added to treat cultured RAW264.7 murine macrophage cells for 6 hrs. Culture supernatants were collected to assay TNFα production by ELISA.

The comprehensive genome analysis of *V. cholerae* 7^th^ pandemic El Tor strain N16961 indicated that it possesses 3,887 protein coding genes out of total 4,009 genes in two chromosomes (http://cmr.tigr.org/tigr-scripts/CMR/GenomePage.cgi?org=gvc). In this work, 3,882 *V. cholerae* proteins were successfully synthesized using an *in vitro* expression system and screened for their ability to produce TNFα, a proinflammatory cytokine, in RAW264.7 murine macrophage cells. The RAW264.7 cell line was chosen because (i) they, as macrophage cells, express a broad spectrum of immune-related receptors and (ii) they grow with established culture stability. Schematic screening procedures are depicted in Fig. 1. Positive protein pools were confirmed using a cut-off that was 1.8 fold above the TNFα levels obtained from the reticulocyte lysate only control treatment. Then, proteins in the corresponding pools were individually synthesized and used to stimulate cultures of RAW264.7 cells. In the final screen using individually synthesized proteins, TNFα production greater than 2.1 fold of the negative control value was considered positive for induction. Out of 3,882 *V. cholerae* proteins, 8 proteins were reproducibly identified to stimulate TNFα production and are listed in Table 1. The positive proteins include a protein of unknown function (protein 3), protein-modifying enzymes (proteins 4, 6, 7, and 8), a lipid-modifying enzyme (protein 5) and two membrane-associated proteins (proteins 1 and 2).

**Table 1:** List of proteins identified to cause RAW264.7 cells to produce TNFα.

Proteins	TNFα (pg/ml)
1. VC1085, Sensor-Histidine Kinase	1430.8, 1150.0
2. VC2283, Sodium-Dependent Transporter	1496.6, 1315.6
3. VC1893, Conserved Hypothetical Protein	1172.2, 1186.0
4. VC2261, Methionine Aminopeptidase	1521.0, 1361.1
**5.** **VC0339, Phosphatidylserine Decarboxylase**	1142.5, 1292.9
6. VC1494, Aminopeptidase N	1253.3, 1539.0
7. VC0556, Glutamate-cysteine Ligase	1177.2, 1232.6
8. VCA0975, ATP-dependent protease LA-related protein	1082.2, 1317.6
No plasmid DNA control	568.0, 531.1

ELISA results from two independent experiments were shown on the right column. For controls, cells were treated with protein synthesis reaction mixtures, in which H_2_O instead of plasmid DNA was added.

**Figure 2:**
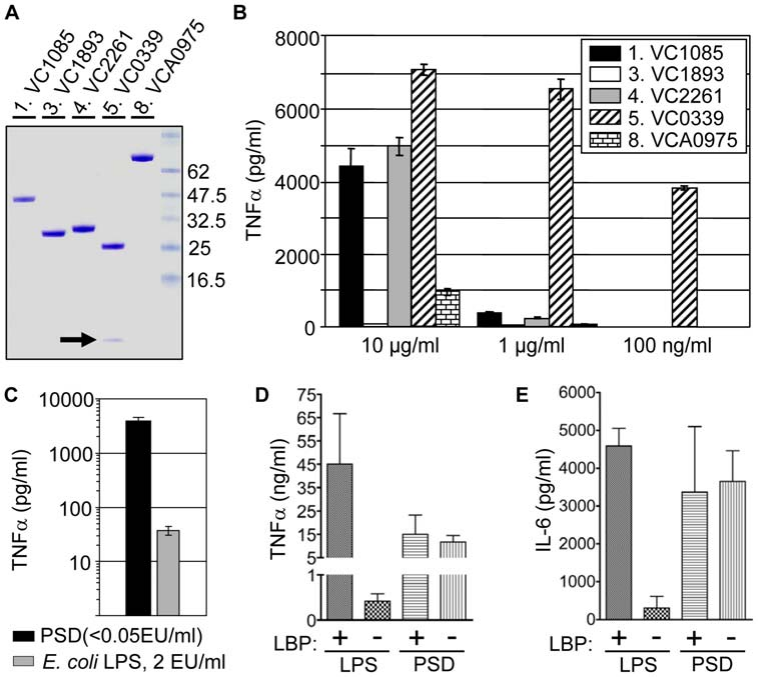
Further verification of screening results using purified recombinant proteins. (A) Purity of the purified proteins in SDS-PAGE. Molecular weight markers (in kDa) are shown on the right lane. One µg of each protein was separated in 4–12% SDS-PAGE. (B) TNFα production of RAW264.7 cells in response to each of five purified proteins. Three different protein concentrations were used to treat cells. Prior to being added to cells, LPS was removed from the protein solution to the level of <0.05 EU/ml. RAW264.7 cells were grown in DMEM plus 10% FCS for overnight and cells were treated with each protein in serum-free DMEM for 6 hrs. (C) TNFα production of RAW264.7 cells in response to the purified PSD (VC0339, 100 ng/ml)) and *E. coli* LPS in 2 EU/ml final concentration. The same *E. coli* LPS was used to make a standard curve for LPS quantification in the PSD sample. (D) Effect of the presence of LBP on TNFα production. RAW264.7 cells were treated with LPS (10 ng/ml) or PSD (100 ng/ml) in serum-free DMEM in the presence or absence LBP (100 ng/ml) for 6 hours. (E) Effect of the presence of LBP on IL-6 production. RAW264.7 cells were treated with LPS (100 ng/ml) or PSD (1 µg/ml) in serum-free DMEM in the presence or absence of LBP (100 ng/ml) for 6 hours.

Although the proteins in Table 1 were identified using appropriate screening controls, the rabbit reticulocyte lysate that was used to drive the *in vitro* protein synthesis reaction contains many unknown materials that may be contaminating in the assay. To further verify the screening result, RAW264.7 cells were treated with purified proteins expressed in *E. coli* BL21 (DE3). Among the eight proteins shown in Table 1, five proteins were successfully purified as His^6^ tagged-recombinant proteins (Fig. 2A). The other three proteins were either not expressed or expressed as insoluble proteins (data not shown). Since RAW264.7 cells are highly sensitive to LPS, LPS was removed from purified proteins down to <0.05 EU/ml as described in Materials and Methods (data not shown). As shown in Fig. 2B, varying doses of the purified recombinant proteins were used to stimulate cultures of RAW264.7 cells. Proteins 1, 4, 5, and 8 induced RAW264.7 cells to produce TNFα, whereas purified VC1893 (Protein No. 3) failed to elicit TNFα production. Interestingly, **p**hosphatidyl**s**erine **d**ecarboxylase (Protein No. 5, VC0339, herein called PSD) stimulated TNFα production even at the lowest concentration tested (100 ng/ml) suggesting that PSD stimulates macrophages with the greatest potency.

To further prove that PSD-induced TNFα production is not due to LPS contamination, we compared the level of TNFα produced in response to the purified PSD and *E. coli* LPS. As shown in Fig. 2C, in response to 2 EU/ml *E. coli* LPS, a level that is >40-fold higher than that detected in the purified PSD, RAW264.7 cells produced only ∼0.7% of the level of TNFα produced by PSD. It has been reported that LPS response, especially at lower concentration of LPS, is enhanced by the presence of LPS-binding protein (LBP), which facilitates the efficient delivery of LPS to CD14 [24],[25]. As expected, the addition of LBP dramatically enhances the production of TNFα (Fig. 2D) and IL-6 (Fig. 2E) in response to LPS. In contrast, pro-inflammatory cytokine production was robust regardless of the presence or absence of LBP in RAW264.7 murine macrophage cells responding to PSD (Fig. 2D and E, right bars). Collectively, these results strongly support that PSD-induced proinflammatory response from stimulated macrophages is not caused by LPS contamination in the purified PSD protein.

PSD is an important enzyme involved in the synthesis of phospholipid bilayer in both eukaryotic and prokaryotic organisms [26],[27],[28]. *V. cholerae* PSD is 285 amino acids long and is produced as an immature proenzyme, which then undergoes autocatalytic cleavage by α, β-elimination. Biologically active mature enzyme thus produced consists of two subunits, a 27.9 kDa β-subunit and a 3.6 kDa α-subunit (black arrow in Fig. 2A) [29].

### PSD-induced proinflammatory response is MyD88-dependent and is more potent than other known TLR ligands in inducing interleukin-6 (IL-6) production from peritoneal mouse macrophage cells

**Figure 3:**
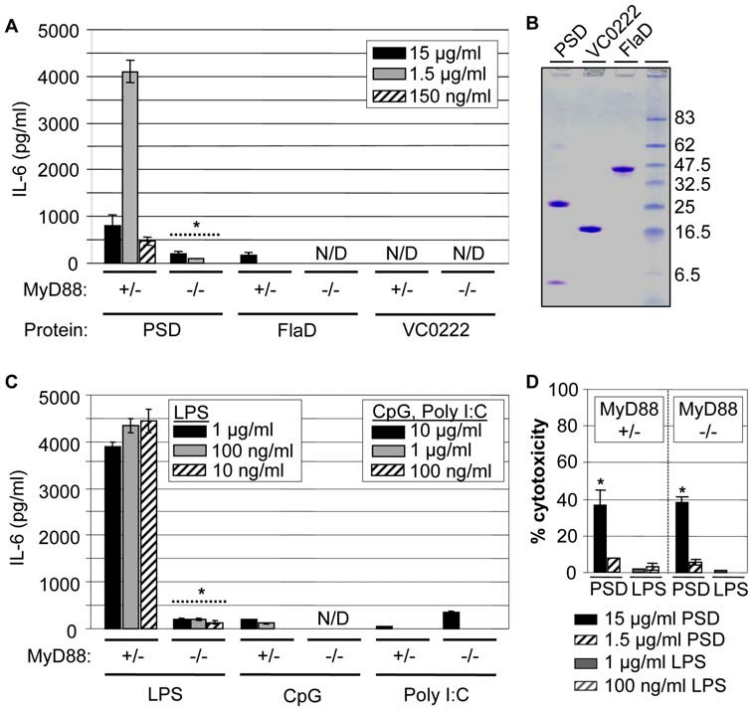
PSD-induced proinflammatory response is MyD88-dependent and PSD, in higher concentration, exerts a cytotoxic effect on peritoneal mouse macrophages. (A) IL-6 production in peritoneal macrophages isolated from MyD88+/− (left) or MyD88−/− (right) mouse in response to three purified *V. cholerae* proteins (PSD, FlaD and VC0222). Cells were treated with the protein in three different final concentrations, 15 µg/ml (black bars), 1.5 µg/ml (gray bars) and 150 ng/ml (hatched bars). Isolation and stimulation of resident murine macrophages were performed as described in Materials and Methods. *p<0.01 vs. IL-6 production in MyD88+/− cells in each corresponding protein concentration. (B) Purity of the protein stimulants was shown in SDS-PAGE. One µg of each protein was separated in 4–12% SDS-PAGE. (C) IL-6 production in peritoneal macrophages isolated from MyD88+/− (left) or MyD88−/− (right) mouse in response to non-protein ligands indicated at the bottom of the graph. Experimental conditions were identical with Fig. 3A. *p<0.01 vs. IL-6 production in MyD88+/− cells in each corresponding protein concentration. (D) LDH activity was measured in the same culture supernatant that was used for IL-6 ELISA. % cytotoxicity was calculated as a relative LDH activity of maximally released LDH by a treatment of 1% triton X-100. *p<0.01 vs. the other treatments.

To gain an insight into the signaling mechanism(s) by which PSD activates mouse macrophages, PSD was used to stimulate the induction of the proinflammatory cytokine, IL-6, in MyD88−/− (Myeloid Differentiation Factor-88) and MyD88+/− macrophages. MyD88 is one of most commonly used adaptor molecules that mediates signal transduction in mammalian innate immune activation [30],[31]. Upon ligand binding, TLRs recruit many downstream signaling molecules via MyD88 to activate NF-κB, which then transcribes genes involved in the production of proinflammatory cytokines [1]. LPS is a known potent stimulator of MyD88-dependent inflammatory pathway and stimulates a high level of IL-6 secretion from MyD88 positive macrophages compared to MyD88 negative macrophages [32]. When treated with PSD or LPS, MyD88 knockout macrophage cells secreted significantly less IL-6 compared to the level of IL-6 detected in MyD88 positive macrophages (Fig. 3A and C) indicating that PSD triggers through a predominantly MyD88-dependent inflammatory signaling cascade similar to that observed with LPS.

To determine the relative strength of PSD as an immunostimulant, adhered primary macrophages were also treated with various doses of other known TLR agonists (Fig. 3A and C). FlaD (VC2143) from flagella and CpG DNA are known TLR agonists that act through a MyD88-dependent pathway [11],[33]. They both induce IL-6 secretion from MyD88 positive macrophages but not from MyD88 negative macrophages. In both cases, the level of secretion is not as great as that observed with either LPS or PSD stimulation (Fig. 3A and C). To further rule out the possibility that the IL-6 production is due to any contaminant that might have been incorporated in PSD sample during purification, another *V. cholerae* protein (VC0222, pantetheine-phosphate adenylyltransferase), which was purified in parallel with PSD and FlaD, was also used as a negative control in this assay. After LPS was removed, the purity of these three proteins was shown by SDS-PAGE (Fig. 3B). We observed that PSD was more potent at eliciting IL-6 production than FlaD, and IL-6 production was not detectable in cells treated with VC0222 (Fig. 3A). Again, this suggests that IL-6 production by PSD is caused by the specific interaction of PSD with macrophage cells and not by any other unknown contaminant in the protein sample such as LPS.

Poly I:C (dsRNA mimic) is a TLR agonist that stimulates innate immune activation through a MyD88-independent pathway [34]. When poly I:C was used to stimulate peritoneal macrophages from MyD88 knockout and positive mice, we observed a higher level of IL-6 (Fig. 3C) and IFN-β (Fig. S1) in MyD88−/− macrophages than in MyD88 intact cells. This suggests that the decrease in IL-6 production from MyD88 knockout macrophages induced by PSD or LPS is not due to a non-specific decrease in ligand-responding capacity of the MyD88 knockout cells.

### PSD, in higher concentration, exerts a cytotoxic effect on macrophages

Interestingly, we detected a decreased level of IL-6 production in MyD88+/− mouse peritoneal macrophages responding to the higher dose of PSD (Fig. 3A, black and gray bars). To determine whether this result is due to PSD-induced cytotoxicity, we measured lactate dehydrogenase (LDH) activity in the cultures of peritoneal macrophage cells. We observed that PSD exerted a cytotoxic effect on MyD88+/−macrophage cells at 15 µg/ml concentration (Fig. 3D, left side) as well as in MyD88−/− macrophage cells (Fig. 3D, right side), suggesting that the PSD-triggered cytotoxicity occurs irrespective of the presence of MyD88. In contrast, LPS-stimulated macrophage cells did not exhibit LDH release in culture supernatants of either MyD88−/− or MyD88+/− cells suggesting that LPS induction of proinflammatory responses is not cytotoxic in nature (Fig. 3D).

### Immunostimulatory activity of PSD is, in part, dependent on its enzymatic activity

**Figure 4:**
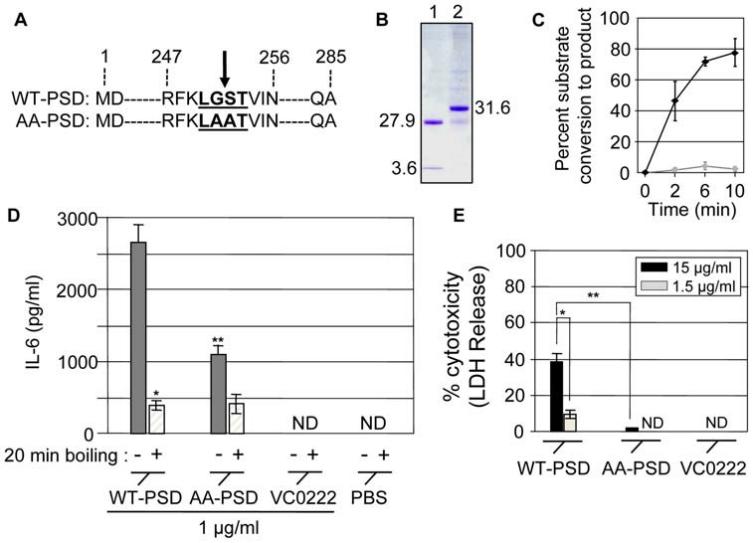
Involvement of the enzymatic activity of PSD in IL-6 production and cytotoxicity. (A) Primary amino acid sequences of WT-PSD and alanine replaced PSD mutant (termed AA-PSD). LGST residue determined to be processed by an autocleavage (shown as a black arrow) was underlined. (B) SDS-PAGE of purified WT-PSD (lane 1) and AA-PSD (lane 2). (C) Enzyme activity assay of the purified proteins (WT-PSD: black line, AA-PSD: gray line). Conversion rate of radiolabeled substrate via the enzymatic activity was displayed as mean±SEM, n = 3. (D) IL-6 production in peritoneal mouse macrophages isolated from BALB/c mice by WT-PSD and AA-PSD (native or heat-inactivated). VC0222 and PBS were used as controls. *p<0.01 vs. IL-6 production by native WT-PSD. **p<0.01 vs. IL-6 production by native WT-PSD. (E) Peritoneal macrophages were treated with three different proteins indicated at the bottom of the graph in two concentrations (15 µg/ml, black bars; 1.5 µg/ml, gray bars) for 15 hrs. LDH activity was measured as in Fig. 3D. *p<0.01 vs. % cytotoxicity by 15 µg/ml WT-PSD. **p<0.001 vs. % cytotoxicity by 15 µg/ml WT-PSD.

To determine whether the proinflammatory response elicited by PSD is mediated by its enzymatic activity, an enzymatically inactive mutant of PSD was constructed and expressed. Biologically active enzyme consists of two subunits as shown in SDS-PAGE gels (Fig. 2A and 3B). Fig. 4A shows primary sequence of *V. cholerae* wild-type PSD and the mutant PSD, in which the wild-type LGST motif (underlined) identified to be the processing site for autocatalytic cleavage [29],[35] has been changed to LAAT in the mutant protein. As expected, the AA-PSD mutant was purified as a single polypeptide (Fig. 4B), and no enzymatic activity was observed in the mutant (Fig. 4C).

When murine peritoneal macrophages were treated with either wild-type or mutant forms of PSD, a reduced level of IL-6 (∼40%) was detected in culture supernatants of cells treated with AA-PSD compared to cells incubated with WT-PSD (Fig. 4C). Furthermore, heat-inactivated WT-PSD, when compared to untreated WT-PSD, elicited significantly reduced IL-6 production in macrophage cells (Fig. 4C). Because the stimulatory activity of LPS is not affected by heat inactivation (data not shown), this results further supports our conclusion that the stimulatory activity of PSD is not due to LPS contamination. IL-6 was not detected in supernatants of cells incubated with the control protein, VC0222 (Fig. 4C) or PBS confirming that our purification protocols effectively removed residual LPS from these recombinant proteins. However, IL-6 production was not completely abrogated by treating macrophages with either the heat-inactivated WT-PSD or AA-PSD suggesting that a linear, nonconformational epitope of PSD is likely recognized by macrophages in this assay.

We then asked if AA-PSD causes the cytotoxic effect in peritoneal macrophages similar to that observed by stimulating cells with WT-PSD (Fig. 3D). As shown in Fig. 4D, LDH release was not detected in cells treated with AA-PSD at the two different concentrations tested suggesting that the cytotoxic effect is most likely due to the biological activity associated with the WT-PSD.

### TLR4 is involved in PSD-induced proinflammatory signal transduction

**Figure 5:**
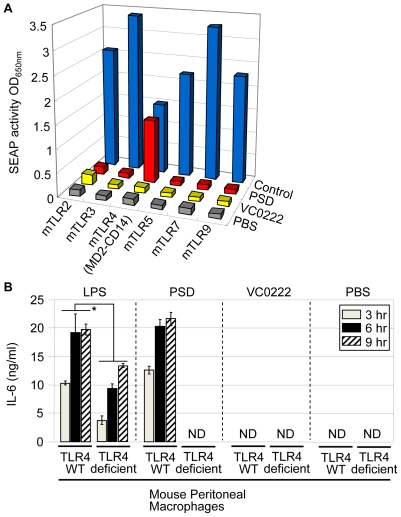
Stimulatory effect of PSD on mouse TLR4. (A) SEAP reporter activity was measured in HEK293 cells transfected with each mouse TLR construct (indicated at the bottom) and plotted as a 3-D bar graph. Cells expressing each TLR were treated with its own control ligand (blue bars), WT-PSD (3 µg/ml, red bars), VC0222 (3 µg/ml, yellow bars) and PBS (gray bars) in two independent cultures. Average activity values were used for plotting. LPS levels in the final preparation in PSD and VC0222 were 0.035 (±0.00191) and 0.027 (±0.0007) EU/ml, respectively. (B) Peritoneal macrophage cells isolated from C3H/HeOuJ (TLR4 WT) or C3H/HeJ (TLR4-deficient) mice were treated with *S. typhimurium* LPS (100 ng/ml), WT-PSD (1.5 µg/ml), VC0222 (1.5 µg/ml) and PBS for 9 hrs and IL-6 level in each treatment was measured after 3 (gray bars), 6 (black bars) and 9 hrs (hatched bars). LPS levels in the final preparation in PSD and VC0222 were 0.035 (±0.00191) and 0.027 (±0.0007) EU/ml, respectively. *p<0.01 vs. IL-6 production from TLR4 intact macrophages in each time point.

Our results shown in Fig. 3A indicated that *V. cholerae* PSD elicits proinflammatory responses in a MyD88-dependent manner. Since most TLRs use MyD88 as a key adaptor protein to recruit downstream signaling molecules [1],[31], we hypothesized that PSD may signal through a TLR to activate innate immune responses and the production of proinflammatory cytokines. To test this hypothesis, HEK293 (human embryonic kidney) cells, which do not express endogenous TLR [36], were transfected with each of the individual TLRs (TLR2, 3, 4, 5, 7, and 9) and assayed for activation by PSD. To ensure full responsiveness to LPS, the plasmid expressing *tlr4* co-transcribes genes encoding CD14 and MD2, which are involved in LPS responses [37]. HEK293 cells were also transfected with a reporter construct in which the expression of secreted alkaline phosphatase (SEAP) is driven from an NF-κB inducible promoter. Appropriate positive controls (Fig. S2) were tested to compare their NF-κB signaling activity with that of PSD. As shown in Fig. 5A, HEK293 cells expressing each TLR responded to its corresponding ligand (blue bars). No cross reactivity was detected in HEK293 cells responding to other control ligands (data not shown). We observed that PSD-induced NF-κB activation was most efficiently detected in HEK293 cells expressing TLR4/MD2/CD14 in comparison to the other TLRs, indicating that PSD likely signals through TLR4 (Fig. 5A).

To further prove the role of TLR4 in PSD signaling, we next compared the IL-6 production from peritoneal macrophages freshly isolated from TLR4 WT (C3H/HeOuJ) and TLR4 hyporesponsive mice (C3H/HeJ) in response to PSD stimulation. Fig. 5B shows the time course of IL-6 production from cells in response to *S. typhimurium* LPS, PSD, VC0222 and PBS. In the presence of LPS, decreased levels of IL-6 production were detected at all time points in TLR4 deficient macrophages compared to levels detected in TLR4 intact cells (Fig. 5B). However, IL-6 production was only reduced by approximately 50% in the TLR4 deficient macrophages after stimulation with LPS suggesting that alternative LPS responding pathways exist in these cells. Indeed, LPS-induced IL-6 production from TLR4-deficient macrophages may also be mediated by TLR2 as previously reported [38],[39]. In contrast, IL-6 production was completely abrogated in PSD-treated TLR4-deficient macrophage cells, while a high level of IL-6 was produced in TLR4 intact cells (Fig. 5B). When we treated the same TLR4 positive or deficient macrophage cells with phosphatidylserine (PS) or phosphatidylethanolamine (PE), that is the substrate or product of PSD, respectively, no proinflammatory response was detected (data not shown). This further suggests that the PSD-mediated IL-6 induction is not due to the lipid substrate or product of PSD. Collectively, these results show that PSD-induced proinflammatory signal transduction is mediated by TLR4 and further supports our conclusion that PSD's TLR4 agonist activity is not the result of LPS contamination.

### PSD has adjuvant activity against a co-administered antigen

**Figure 6:**
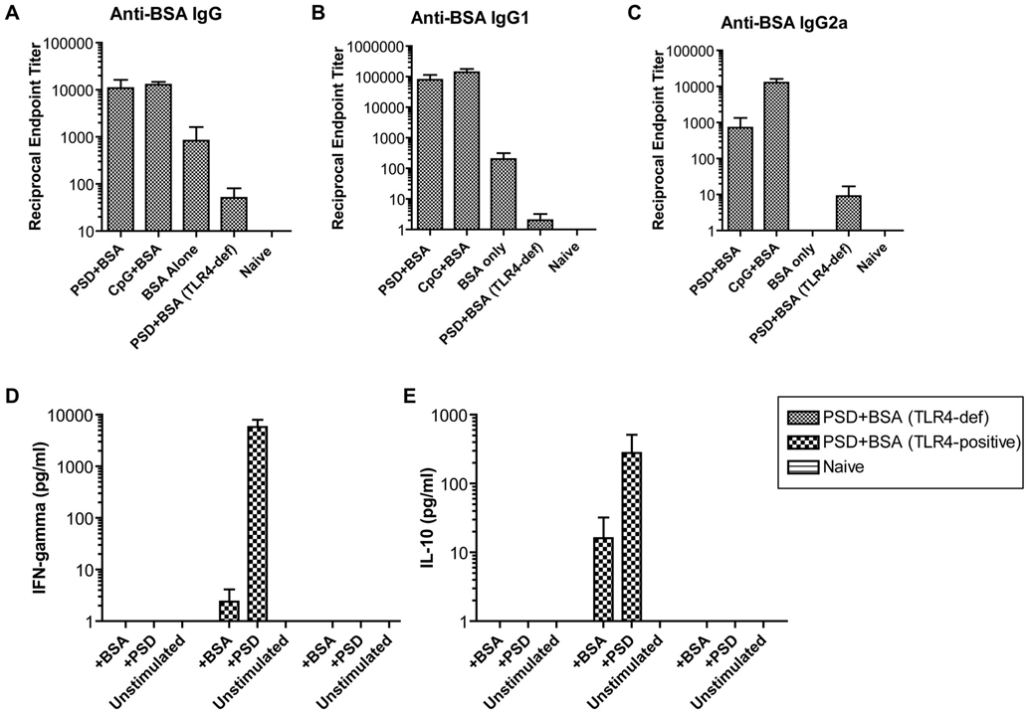
PSD is an adjuvant that induces mixed Type 1 and Type 2 antigen-specific immune responses. (A) Anti-BSA IgG responses following immunization with BSA in the presence or absence of PSD or CpG as adjuvants show enhanced IgG responses in serum from animals immunized with PSD compared to serum from mice immunized with BSA alone. (B and C) IgG1 and IgG2a isotype characterization of anti-BSA humoral responses following immunization with BSA in the presence of PSD or CpG as adjuvants. Both Type 2 IgG1 (B) and Type 1 IgG2a (C) antigen-specific responses are enhanced when PSD was coadministered with BSA. (D and E) PSD increases BSA-specific Type 1 and Type 2 cytokine responses. Antigen restimulation assays using splenocytes from PSD+BSA immunized animals with intact TLR4 demonstrated increased Type 1 IFNγ (D) and Type 2 IL-10 (E) cytokine responses when restimulated with BSA or PSD.

Using this high throughput screen, we have identified PSD as a TLR4 agonist that signals through a MyD88-dependant pathway. One of the hallmarks of TLR agonists is that they induce innate immune responses that can influence and shape adaptive immunity. Because of this property, many TLR agonists are used as adjuvants enhancing the adaptive immune response against co-administered antigens [40]. To determine if PSD can also exert an adjuvant effect, groups of mice were immunized with the inert antigen BSA alone or in the presence of either PSD or CpG as adjuvants. Mice that received BSA in conjunction with PSD showed enhanced anti-BSA antibody responses compared to mice that were immunized with BSA alone or naïve animals (Fig. 6A). This enhanced effect on the anti-BSA immune response was similar to that observed when mice were immunized with a known TLR agonist, CpG [41]. This adjuvant effect exerted by PSD was due to TLR4 activation since TLR4-deficient mice immunized with PSD in conjunction with BSA demonstrated a significant decrease in the anti-BSA immune response compared to when TLR4 intact mice were immunized (Fig. 6A). These data suggest that PSD is a bacterial protein that acts as an adjuvant against a co-administered antigen, BSA and further proves the role of TLR4 in PSD-induced signaling pathway *in vivo*.

Monitoring antibody isotypes generated following immunization with a particular adjuvant can reflect the balance of Th1 or Th2 responses induced. Th1 responses favor the production of IgG isotypes such as IgG2a and Th2 responses favor the production of isotypes such as IgG1 [42]. Isotype characterization of serum from mice immunized with BSA in the presence of both PSD and CpG demonstrated elevated levels of both BSA-specific IgG1 and IgG2a compared to serum from mice that received BSA alone (Fig. 6B and C). In contrast, immunization with BSA alone induced specific IgG1 but not IgG2a responses. These results suggest that PSD acts as an adjuvant that not only enhances the default Type 2 (IgG1) humoral immune response, but also induces IgG2a, a hallmark of Type 1 immunity, against a co-administered antigen. Consistent with results in Fig. 6A, significantly decreased levels of both anti-BSA IgG1 and IgG2a were detected in TLR4-deficient mice.

To determine if cellular responses mirrored the humoral responses, we determined cytokine profiles from antigen restimulated splenocytes. We isolated single cell suspensions of splenocytes from immunized and naïve animals and plated them into 24 well plates in the presence or absence of the BSA antigen or PSD adjuvant. In this assay, the splenocytes from immunized animals will recognize the antigen and start proliferating and secreting cytokines in response. Consistent with isotyping results (Fig. 6B and C), BSA- and PSD-restimulated splenocytes from TLR4-intact mice immunized with PSD+BSA secreted IFN-gamma, a Type 1 cytokine (Fig. 6D) and IL-10, a Type 2 cytokine (Fig. 6E). Antigen-restimulated splenocytes from TLR4-deficient mice immunized with PSD+BSA and naive animals failed to secrete detectable Type 1 or 2 cytokines. Collectively these data show that PSD serves as an adjuvant which enhances both humoral and cell-mediated arms of the immune response against a co-administered antigen.

## Discussion

In this study, we show that *V. cholerae* phosphatidylserine decarboxylase is capable of stimulating innate immune effector cells (macrophages) to secrete proinflammatory cytokines, a hallmark of the innate immune response. PSD was identified in a high-throughout Expressed Protein Screen for Immune Activators (EPSIA). EPSIA provides an approach to screening the entire protein repertoire of an infectious organism for agonists of immunological responses that can be assayed using appropriate eukaryotic reporter cell lines. Our successful application of EPSIA to the discovery of a novel TLR agonist can be attributed to following contributions; (i) the use of a well-established murine macrophage cell line RAW264.7 provided cytokine induction reproducibility and thus, minimized batch to batch variations, (ii) efficient *in vitro* protein synthesis was achieved using the rabbit reticulocyte lysate (RRL) expression system, (iii) LPS, to which RAW274.7 cells are highly sensitive, was successfully removed from plasmid DNA and purified recombinant proteins by ion-exchange column purification and detergent extraction method, respectively and finally, (iv) the control treatment containing RRL mixture, but no plasmid DNA, elicited only a basal level of proinflammatory response from RAW264.7 cells.

The most convincing evidence that *V. cholerae* PSD may play a role as an immunostimulatory protein stems from the results demonstrated in Fig. 2. Purified PSD elicited the strongest TNFα production at concentration as low as 100 ng/ml, while the other three proteins stimulated RAW264.7 cells only at the highest working concentration (10 µg/ml). The experiments using MyD88−/− peritoneal macrophages led to the observation that the PSD-induced proinflammatory response may be mediated by an innate immune signaling pathway. Like LPS, PSD stimulated a MyD88-dependent signaling mechanism to produce IL-6 (Fig. 3A). IL-6 production in the freshly isolated peritoneal macrophages also indicates the *in vivo* relevance of the PSD-induced host proinflammatory responses.

Bioinformatic analysis indicates that almost all bacterial species in the public database possess a gene encoding for PSD. In addition, the gene encoding PSD cannot be inactivated in *V. cholerae* [43]. This suggests that PSD is likely an essential enzyme for bacterial viability and thus, could be a conserved target for detection by the innate immune system. Interestingly, PSD is also present in mammalian cells and sequence alignment between eukaryotic and prokaryotic PSD suggests that eukaryotic PSD are also processed by autocatalytic cleavage using the same LGST motif [44]. Mouse PSD displays 31.7% sequence identity with *V. cholerae* PSD while the latter displays 60% sequence identity with *E. coli* PSD. It is not known whether the divergence in sequence between eukaryotic and prokaryotic PSD is sufficient to block its TLR agonist activity. Importantly, PSD in mammalian cells resides predominantly in mitochondria [45],[46]. This may provide some degree of compartmentalization, where innate immune cells may not detect circulating levels of endogenous PSD unless host cells lyse and release mitochondrial contents. It is interesting that disruption of mitochondria is a hallmark of processes such as apoptosis [47] and thus, mitochondrial PSD may play a role in amplification of inflammatory responses occurring as a result of bacterial or viral replication within and lysis of host cells.

We were also intrigued by the observations that (i) PSD at its highest concentration (15 µg/ml) was cytotoxic to both MyD88+/− and MyD88−/− macrophages (Fig. 3D), and (ii) this cytotoxicity was not detected when the non-functional alanine-substituted PSD mutant (AA-PSD) was used to stimulate cells (Fig. 4D). This suggests that PSD triggers a MyD88-dependent proinflammatory signaling up to a certain threshold, after which PSD induces a non-specific cytotoxicity to host macrophage cells likely due to its enzymatic activity. Further study is necessary to precisely determine whether cytotoxic effect imposed on macrophages by PSD is mediated by apoptosis or random necrosis.

TLR screening of transfected HEK293 cells and antigen stimulation of TLR4-deficient macrophages clearly identified TLR4 as a mediator of the PSD-induced proinflammatory signaling pathway. The gene encoding TLR4 in C3H/HeJ has a point mutation, which results in an amino acid substitution from proline to histidine in the intracellular domain [48]. This amino acid residue change was found to be crucial for the inhibition of binding of MyD88 to downstream signaling molecules [48]. Our results in Fig. 4 demonstrated that the enzymatic activity of PSD appears to be involved, at least in part, in the proinflammatory signaling. However, more detailed experiments are necessary to decipher the precise molecular mechanisms of the bacterial PSD acting on host cells. Recent evidences indicate that upon ligand binding, TLR4 is recruited to a specific domain in the plasma membrane, called the lipid raft [49],[50]. Because PSD is an enzyme that possesses the ability to modify the phospholipid bilayer, we postulate that (i) PSD may directly interact with TLR4 to transduce the proinflammatory signaling or (ii) PSD acting on host cell membrane may affect the interaction between TLR4 and lipid rafts.

Our data also suggest that the activation of macrophages by PSD may be due not only to the enzymatic activity of PSD, but also a binding determinant of PSD that interacts directly with the macrophage, presumably through TLR4. There may be a difference in the accessibility of a binding determinant on PSD, where the binding determinant is more accessible in the active cleaved enzyme than the mutant PSD which effectively remains in the inactive pro-enzyme conformation.

TLR agonists activate innate immune cells to secrete cytokines and more efficiently process and present antigens, which in turn, stimulates robust and effective adaptive immune response [21],[51]. Similarly, we observed that *V. cholerae* PSD exhibited adjuvant activity against a co-administered inert antigen BSA (Fig. 6). PSD stimulated enhanced antibody responses against BSA similar to that observed when a known TLR agonist, CpG was used as an adjuvant compared to when mice were immunized with BSA alone. Isotype characterization of the humoral response and the assaying the cytokine profiles from antigen-restimulated splenocytes following immunization with PSD showed that PSD elicits a mixed Type 1 cell-mediated response in addition to the default Type 2 humoral response. Moreover, both of these responses are enhanced relative to immunization with BSA in the absence of PSD as an adjuvant.

This study identifies *V. cholerae* PSD as a novel bacterial protein that stimulates the host innate immune system, but it still remains unclear (i) whether PSD, as an inner membrane protein, plays a direct role in modulating host innate immune system during a dynamic *in vivo* infection, and (ii) if so, how much and when PSD is released into the environment from invading bacterial cells. Bacterial infection is a complex process, during which bacterial cells are stressed by a number of factors including encountering harsh host immune responses and nutrient deficiency. Thus, it is expected that a subpopulation of invading bacterial cells are lysed during infection, releasing their intracellular components. Interestingly, spontaneous cell death was observed in bacterial biofilms, presumed to be the major mode of bacterial growth in host [52],[53]. Therefore, it is likely that our innate immune system may have been evolved to target intracellular components, such as PSD and previously identified bacterial CpG DNA [10].

In summary, we utilized a proteome-wide screening technique called EPSIA to identify a novel bacterial immunostimulatory protein. Phosphatidylserine decarboxylase (PSD) was shown to activate the host macrophage cells and results provided in this work represent a previously undescribed interaction between host immune system and a bacterially conserved protein. The activity of PSD was further shown to have utility in stimulating immune responses to bystander antigens that were co-administered to animals with PSD as an adjuvant. Thus, EPSIA is a new tool for identifying microbial proteins which are recognized by the innate immune system and may therefore provides an exciting novel approach to identifying antigens and more effective vaccine adjuvants.

## Materials and Methods

### Ethics statement

The methods for animal experimentations were approved by the Harvard Institutional Animal Care and Use Committee (IACUC).

### Screen of the proteome library

*V. cholerae* proteome library was prepared as described in our recent publication [23]. *In vitro* protein synthesis was performed using the TnT® coupled reticulocyte lysate system kit (Promega Cor., Madison, WI) following the manufacturer's instructions. In the primary screening, proteins were synthesized as a pool in each well of 96-well plate. For the secondary screening, proteins in each pool that triggered the TNFα production in RAW264.7 cells were individually synthesized and screened for their activity to produce TNFα. Supernatants were collected after 6 hr incubation and assayed for secreted TNFα by cytokine ELISA (BD Pharmingen). RAW264.7 cells were seeded at 2×10^6^ cells/ml in a 100 µl volume and grown for overnight before being treated with proteins. Cells were grown in DMEM containing 10% FBS at 37°C in a humidified 5% CO_2_ incubator.

### Protein purification and construction of mutant PSD

For recombinant protein production, the encoding genes were PCR-amplified and positionally cloned into pET21b (Novagen). The resulting plasmid was then transformed into *E. coli* BL21 (DE3). 1 mM IPTG was used to induce overexpression and recombinant His-tagged proteins were purified using a Ni-NTA agarose (Qiagen, Valencia, CA). The purity of purified protein was assessed by SDS-PAGE.

QuickChange® site-directed mutagenesis kit (Stratagene, Inc., La Jolla, CA) was used to introduce point mutation (AA replacement). Enzyme activity assay was carried out following the previously published protocols [54],[55].

### LPS removal and cytotoxcity assay

LPS removal and LPS detection assay was performed using EndoClean™ Endotoxin Removal Kit (Biovintage, Inc., San Diego, CA) and Endotoxin detection kit (Cambrex Corp., East Rutherford, NJ), respectively. For cytotoxicity assay, lactate dehydrogenase (LDH) was measured using a spectrometric assay kit (Biovision Inc., Mountain View, CA) following manufacturer's instructions.

### Effect of LBP on the production of proinflammatory cytokines

RAW cells were seeded at 2×10^6^ cells/ml in 96-well tissue culture plates overnight in DMEM containing 10% FBS. The next day, cells were washed 3 times with PBS and incubated for 2 hours with serum-free media. PSD and LPS at the indicated concentrations were added to cells in fresh serum-free media alone or to serum-free media containing 100 ng/ml LPS-binding protein (R&D Systems). Cells were then incubated at 37 C, 5% CO2 for 6 hours. Supernatants were collected and assayed for TNFa and IL-6 by capture ELISA.

### TLR screening

TLR stimulation was tested by assessing NF-κB activation in HEK293 cells expressing a given TLR. The activity of sample is tested on six different mouse TLRs: TLR2, 3, 4, 5, 7 and 9 (Invivogen, San Diego, CA). HEK293 cells were also transfected with a reporter construct, in which the secreted alkaline phosphatase (SEAP) is expressed by a NF-κB inducible promoter. TLR stimulation in the screening is tested by assessing NF-κB activation in the HEK293 cells expressing a given TLR. After a 16–20 hr incubation, SEAP activity was monitored by measuring the OD_650_ on a Beckman Coulter AD 340 C Absorbance Detector.

### Mice

Five to seven week old female BALB/c mice were purchased from Charles River. Five to seven week old C3H/HeJ and C3H/HeOuJ mice were purchased from Jackson Labs. MyD88−/− and MyD88+/− mice were from an in-house colony at Harvard Medical School. All mice were housed using sterile set-up and were allowed a one week acclimatization period before initiation of experiments.

### Isolation and stimulation of resident murine macrophages

Mice were euthanized and injected with 10 ml of cold PBS into the peritoneum. Resident cells were then lavaged out of the peritoneum and pooled. Red blood cells were lysed using a hypotonic buffer and the cells were resuspended in complete RPMI-10 media. These cells were used to seed 96-well plates at 2×10^6^ cells/ml in a 100 µl volume and allowed to adhere for 2–3 hours at 37 °C, 5% CO_2_. Unbound cells were removed by washing the plate 3 times with PBS. Adhered cells were incubated with varying doses of stimulants as indicated in 100 µl volume of serum-free media for 15 hrs. Supernatants were collected at the times indicated and assayed for secreted IL-6 by cytokine ELISA (BD Pharmingen).

### Immunization protocol

Groups of four to five BALB/c or C3H/HeJ mice were immunized three times at biweekly intervals intraperitoneally (IP) with 10 µg BSA in the presence or absence of 5 µg of PSD. Other groups of BALB/c mice were similarly immunized with 10 µg BSA alone or in conjunction with 10 µg CpG DNA. Two weeks after the last immunization, all animals were sacrificed. Blood was collected from each mouse by cardiac puncture, and serum was obtained for the determination of BSA-specific responses by ELISA. Spleens were collected from each mouse to perform antigen restimulation assays.

### Antigen restimulation assays

Spleens from immunized or naïve animals were homogenized through a sterile cell dissociation sieve, pelleted and resuspended in RPMI 1640 (1% FBS, 2% Ab/Am). Splenocytes were isolated using density centrifugation with Histopaque-1119, washed, and resuspended in complete RPMI 1640 (10% FBS, 2% Ab/Am, 2 mM L-Glutamine, 50 µM β-mercaptoethanol, 1 mM sodium pyruvate, and 1 mM MEM non-essential amino acids) containing 0.4 ng/ml of IL-2. Purified mononuclear cells were counted and 2.5×10^6^ cells were added to the wells of a 24-well tissue culture plate. Cells were restimulated with 1 µg BSA or left unstimulated. Supernatants were collected at 3, 4, and 5 days and kept at −20°C until assayed for Type 1 (IFN-gamma) and Type 2 cytokines (IL-10) by cytokine ELISA (BD Pharmingen).

### Antibody assays

Serum samples from groups of immunized mice were analyzed for antigen-specific IgG by ELISA. Immune sera was diluted in PBS-0.05% Tween 20 and added to microtiter plates precoated overnight with 1 µg BSA per well. Rabbit anti-mouse IgG, IgG1, or IgG2a conjugated to alkaline phosphatase was used to determine serum anti-BSA levels. Plates were visualized by the addition of p-nitrophenol substrate to each well. Reactions were stopped with 2 N NaOH and the absorbance at 405 nm was determined on a spectrophotometer. Data are reported as reciprocal endpoint titer, with the cutoff calculated as two standard deviations above the mean of the negative control.

### Statistical analysis

Data are expressed as mean±standard error of the mean. An unpaired Student's *t* test was used to analyze the data. A *P* value of <0.05 was considered statistically significant.

## Supporting Information

Figure S1 List of positive controls that were used to stimulate each TLR.
(0.89 MB TIF)

Figure S2 IFN-β production in peritoneal macrophages isolated from MyD88+/− (left) or MyD88−/− (right) mouse in response to Poly I:C. Experimental conditions were identical with those described in the legend to Fig. 3C.
(0.13 MB TIF)
