# Supplementary figures and images for: Vibrio cholerae Proteome-Wide Screen for Immunostimulatory Proteins Identifies Phosphatidylserine Decarboxylase as a Novel Toll-Like Receptor 4 Agonist
Source: PLoS Pathog. 2009 Aug 21;5(8):e1000556. doi: 10.1371/journal.ppat.1000556 (PMC2722020; doi:10.1371/journal.ppat.1000556)

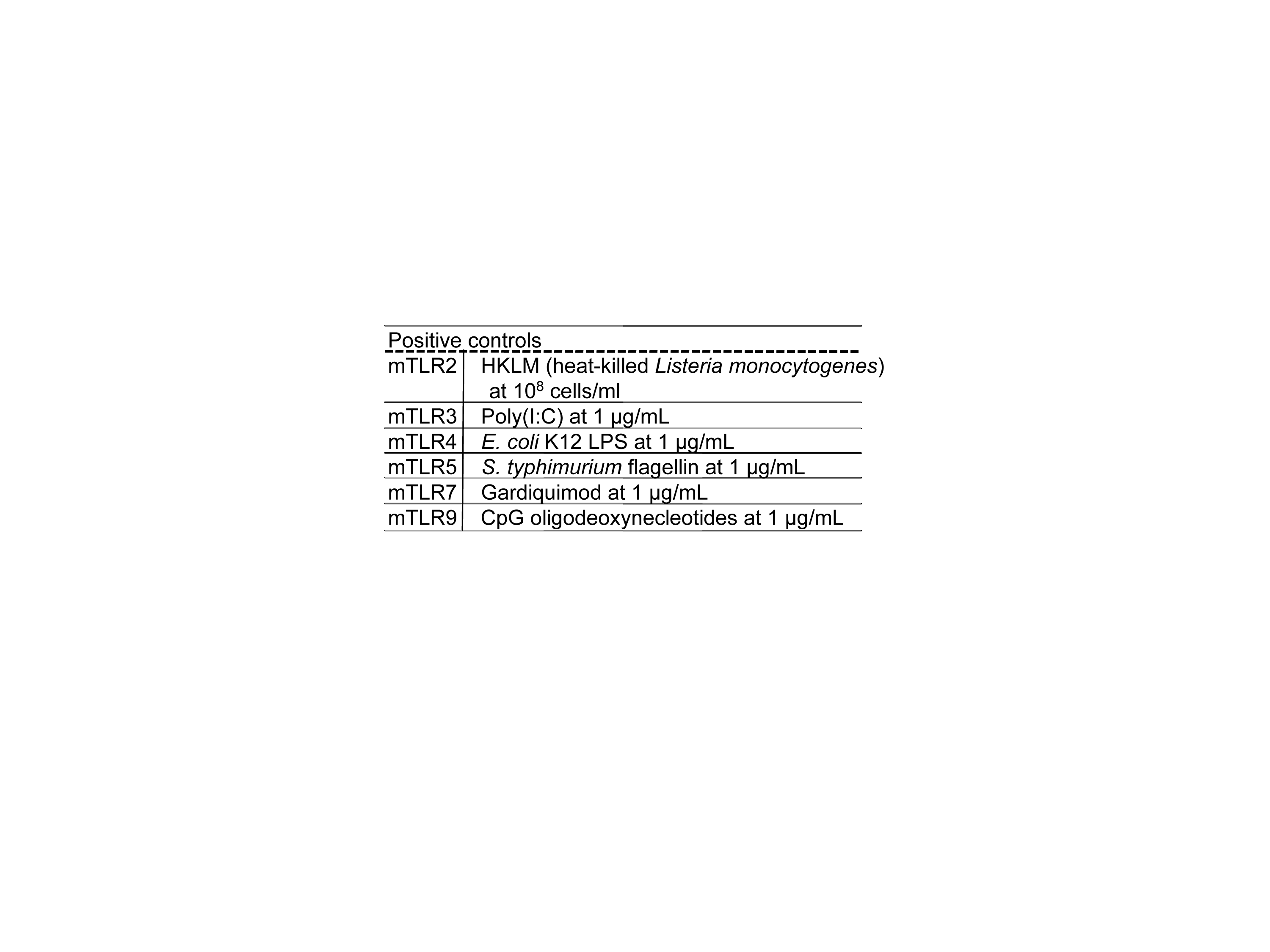

Supplement: Figure S1 — List of positive controls that were used to stimulate each TLR. (0.89 MB TIF) [file ppat.1000556.s001.tif]

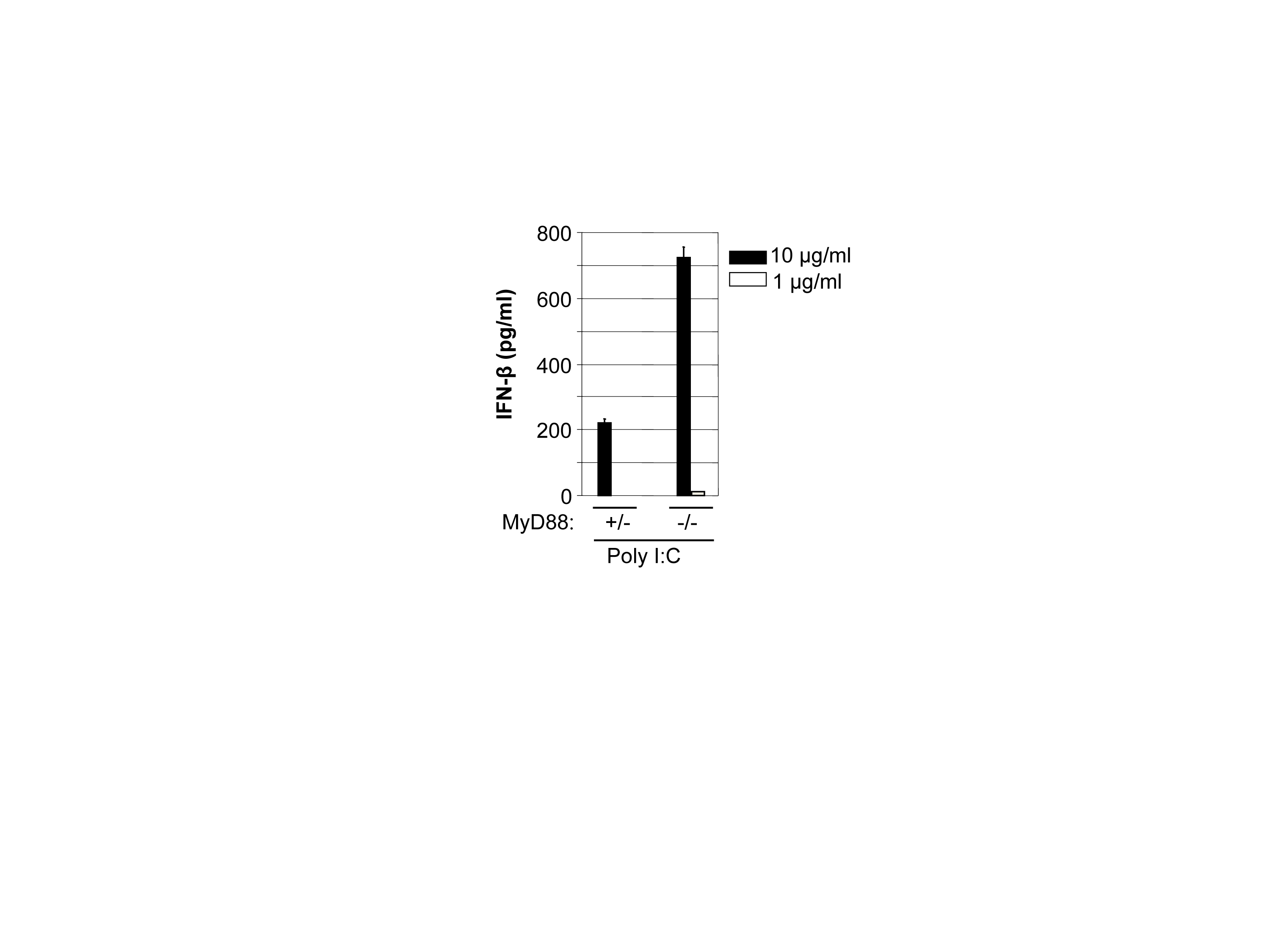

Supplement: Figure S2 — IFN-β production in peritoneal macrophages isolated from MyD88+/− (left) or MyD88−/− (right) mouse in response to Poly I:C. Experimental conditions were identical with those described in the legend to Fig. 3C. (0.13 MB TIF) [file ppat.1000556.s002.tif]
